# The effects of sandostatin (Octreotide, SMS 201-995) infusion on splanchnic and hepatic blood flow in an experimental model of hepatic metastases.

**DOI:** 10.1038/bjc.1992.80

**Published:** 1992-03

**Authors:** D. M. Hemingway, S. A. Jenkins, T. G. Cooke

**Affiliations:** University Department of Surgery, Glasgow Royal Infirmary, UK.

## Abstract

Manipulation of hepatic blood flow may improve drug delivery to hepatic tumour. Somatostatin and its long acting analogues are known to elicit effects upon hepatic and splanchnic blood flow in experimental animals and patients with portal hypertension. This study investigates the effects of SMS 201-995 (sandostatin) infusion on hepatic, splanchnic and tumour blood flow in an experimental model of liver metastases. Hepatic tumour was induced by the intraportal inoculation of 10(6) HSN sarcoma cells and blood flow measured using the dual reference microsphere method before and after infusion of SMS 201-995. There was a significant decrease in hepatic arterial flow and a significant increase in the tumour:liver blood flow ratio associated with a marked reduction in blood flow to normal hepatic parenchyma. Portal venous inflow and tumour blood flow were not significantly affected. SMS 201-995 infusion may lead to preferential delivery of concomitantly injected cytotoxic drugs to hepatic tumour. In addition, the reduction in growth of hepatic tumour may be due to a reduction in nutritive, arterial blood flow to hepatic tumour.


Br. J. Cancer (1992), 65, 396-398 © Macmillan Press Ltd., 1992

### The effects of sandostatin (Octreotide, SMS 201-995) infusion on splanchnic and hepatic blood flow in an experimental model of hepatic metastases

D.M. Hemingway, S.A. Jenkins & T.G. Cooke

*University Departments of Surgery, Glasgow Royal Infirmary and Royal Liverpool Hospital, UK.*

**Summary** Manipulation of hepatic blood flow may improve drug delivery to hepatic tumour. Somatostatin and its long acting analogues are known to elicit effects upon hepatic and splanchnic blood flow in experimental animals and patients with portal hypertension. This study investigates the effects of SMS 201-995 (sandostatin) infusion on hepatic, splanchnic and tumour blood flow in an experimental model of liver metastases.

Hepatic tumour was induced by the intraportal inoculation of $10^6$ HSN sarcoma cells and blood flow measured using the dual reference microsphere method before and after infusion of SMS 201-995.

There was a significant decrease in hepatic arterial flow and a significant increase in the tumour:liver blood flow ratio associated with a marked reduction in blood flow to normal hepatic parenchyma. Portal venous inflow and tumour blood flow were not significantly affected.

SMS 201-995 infusion may lead to preferential delivery of concomitantly injected cytotoxic drugs to hepatic tumour. In addition, the reduction in growth of hepatic tumour may be due to a reduction in nutritive, arterial blood flow to hepatic tumour.

Up to 60% of patients with colorectal cancer develop liver metastases which are rarely surgically resectable (Finlay *et al.*, 1982) and systemic or regional chemotherapy is often used to treat these patients (Kemeney *et al.*, 1987). The extraction of regionally delivered drug by the liver is inversely proportional to hepatic blood flow (Sigurdson *et al.*, 1986) and as many hepatic tumours are hypovascular this may limit delivery of the chemotherapeutic drug to tumour and may, in part, explain the relatively poor response rates to chemotherapy.

Manipulation of hepatic blood flow by vasoactive agents may therefore improve extraction of drug by the liver. In addition, an alteration in the relative blood flow distribution between the normal liver parenchyma and hepatic tumour may also improve drug delivery to hepatic tumour.

Somatostatin and its long acting analogue sandostatin (Octreotide, SMS 201-995), are known to elicit effects upon hepatic and splanchnic blood flow in experimental animals and patients with cirrhosis and portal hypertension (Keller *et al.*, 1978; Price *et al.*, 1985; Sonnenbert *et al.*, 1981). We have therefore investigated the effects of SMS 201-995 infusion on hepatic, splanchnic and tumour blood flow in an experimental model of hepatic metastases.

### Methods

### *Tumour induction*

HSN sarcoma cells were grown in Dulbecco's modified Eagles Medium (Sigma, UK) supplemented with 10% foetal calf serum at 37°C in an incubator. Hepatic tumour was induced by the intra-portal inoculation of $10^6$ HSN sarcoma cells, trypsinised from a confluent monolayer, in male Hooded Lister rats. Our previous studies have demonstrated that discrete hepatic tumour is present 3 weeks after the inoculation of these tumour cells (Hemingway *et al.*, 1991*b*).

Correspondence: T.G. Cooke, University Department of Surgery, Glasgow Royal Infirmary, Glasgow G31 2ER, UK.
Received 29 July 1991; and in revised form 12 November 1991.

### *Hepatic haemodynamics*

Organ blood flow, before and after the infusion of SMS 201-995 was measured using a dual microsphere technique. In brief, 10 tumour bearing rats, 250 g weight, were anaesthatised with intraperitoneal sodium pentobarbitone. One hundred thousand $^{57}$Co microspheres (Nentrac, Dupont, Germany) were suspended in normal saline with 0.01% Tween in a volume of 0.3 ml and injected over 29 s via a cannula (Portex, Hythe, UK) screened into position in the left ventricle using a Siemens Image Intensifier (Siemens, Germany) via the right carotid artery. A reference sample of blood was withdrawn at a rate of 1 ml min$^{-1}$ from the right femoral artery starting 10 s before and continuing for 40 s after the microsphere injection. The withdrawal rate was constant at 1 ml min$^{-1}$. Arterial blood pressure was measured using a strain gauge transducer and pen recorder attached to a cannula in the left femoral artery. The animals received an intravenous bolus of SMS 201-995, 4 $\mu$g kg$^{-1}$, in a volume of 80 $\mu$l, followed by a continuous infusion at a rate of 4 $\mu$g kg$^{-1}$ h$^{-1}$ in a volume of 0.2 ml min$^{-1}$ for 10 min. At the end of the infusion the blood flow measurements were repeated by a further intraventricular bolus injection of $^{51}$Cr microspheres as described previously for $^{57}$Co, and a second reference sample obtained. Five minutes later the animals were humanely killed, the organs were removed, weighed, placed in vials and counted on a well gamma counter along with the reference samples of blood. Counts were corrected for Compton effect down scatter into the $^{51}$Cr channel.

### *Analysis of data*

Organ blood flow was calculated using the method of McDevitt and Nies (1976).

$$\text{Organ blood flow} = \frac{\text{activity in each sample (c.p.m.)}}{\text{activity in reference sample (c.p.m.)}} \times \text{withdrawal rate}$$

Hepatic arterial flow was determined from the counts in the liver. Portal venous inflow was determined from counts in the splanchnic organs draining into the portal vein and hepatosplanchnic flow as the sum of hepatic arterial and portal venous inflow. Hepatic tumour was carefully dissected from the surrounding normal hepatic parenchyma, weighed and counted separately, to enable blood flow to tumour and normal liver to be calculated. Each animal acted as its own control since we have previously demonstrated that infusion of saline at a rate of 0.2 ml $min^{-1}$ for 10 min into tumour bearing rats does not significantly affect blood flow (Hemingway *et al.*, 1991*a*). Animals were discarded from further analysis if blood flow between the right and left kidneys differed by greater than 10% since this indicates inadequate ventricular mixing of microspheres, and if any organ contained less than 400 particles to ensure adequate counting statistics. The hepatic replacement by tumour was calculated as the weight of tumour tissue as a percentage of the total liver weight. The effect of SMS 201-995 infusion on hepatic blood flow was compared using the non-parametric Wilcoxons paired rank sum test and the data expressed as medians (interquartile ranges).

### Results

### *Percentage hepatic replacement*

All animals inoculated with tumour cells developed overt hepatic tumours which were distributed throughout the hepatic parenchyma. The median percentage hepatic replacement by tumour was 15% by weight (range 7% to 30%).

### *Systemic blood pressure*

SMS 201-995 had no significant effect upon systemic arterial blood pressure (110(10) mmHg before infusion, 105(15) mmHg after infusion).

### *Organ blod flow*

Infusion of SMS 201-995 resulted in a significant ($P$ = 0.025) decrease in hepatic arterial flow from 4.55 ± 4.24 before to 1.95 (0.97) ml $min^{-1}$ (Table I). However, portal venous inflow was not significantly altered from its pre-infusion rate (Table I), but hepatosplanchnic flow did fall significantly ($P$ = 0.032) from 9.7(8.77) ml $min^{-1}$ to 7.0 (1.99) ml $min^{-1}$ after SMS infusion (Table I). Tumour blood flow was reduced from 0.28(0.18) ml $min^{-1}$ $g^{-1}$ to 0.10(0.08) although the reduction was not statistically significant. In contrast, blood flow to normal hepatic parenchyma was significantly reduced ($P$ = 0.014) from 0.37(0.28) ml $min^{-1}$ $g^{-1}$ to 0.16(0.09) ml $min^{-1}$ $g^{-1}$. The tumour: liver blood flow ratio was significantly increased ($P$ = 0.042) from 0.55(0.47) to 0.75(0.62) (Table I).

### Discussion

We have previously demonstrated that there are significant alterations in liver blood flow in the presence of hepatic tumour (Hemingway *et al.*, 1991*b*). In the present study using rats with hepatic tumour derived from the intraportal inoculation of HSN sarcoma cells hepatic arterial flow contributed almost 50% of the total liver blood flow before SMS 201-995 infusion, compared with its usual 26% in normal animals. These results therefore confirm our previous observations that the growth and development of hepatic tumour is associated with marked changes in hepatic haemodynamics. In addition, these tumours were hypovascular with a mean tumour: liver blood flow ratio of 0.55: 1.

Whilst there are some conflicting reports on the effects of somatostatin and SMS 201-995 in experimental animals and in man, the consensus of opinion is that in portal hypertension, the naturally occurring hormone and its synthetic analogue have some effects (Kleber *et al.*, 1988; Kravitz *et al.*, 1988; Jenkins *et al.*, 1985*a*; 1985*b*). For example, previous studies in cirrhotic patients have demonstrated that somatostatin at a dose of 250 μg $h^{-1}$ reduces total hepatic blood flow (Keller *et al.*, 1978). Similarly, studies in dogs have demonstrated that somatostatin infusion reduces portal venous inflow by up to 30% (Price *et al.*, 1985). Vasoactive agents can have profound effects upon hepatic blood flow in man and animals with hepatic tumour (Hemingway *et al.*, 1991*c*; Sasaki *et al.*, 1985). This is confirmed in this study which clearly demonstrates a significant reduction in both hepatic arterial flow and results in a decrease in total hepatosplanchnic flow when SMS 201-995 was infused. The reduction in hepatic arterial flow was associated with a rise in the tumour:liver blood flow ratio which was due predominantly to a fall in the arterial supply to the surrounding normal hepatic parenchyma, with no significant change in the measured tumour blood flow. This confirms the observations of Mattson *et al.* (1977) who reported that the blood vessels of tumours were immature, lacking muscular or nervous elements, and thus unable to respond to vasoactive agents.

**Table I** The effects of SMS 201-995 on hepatic arterial flow, portal venous inflow, hepato-splanchnic flow, liver and tumour flow and tumour: liver blood flow ratio in rats with overt HSN hepatic tumours

| *Animal* | *Hepatic arterial flow ml $min^{-1}$* | *Portal venous flow ml $min^{-1}$* | *Hepato splanchnic flow ml $min^{-1}$* | *Liver flow ml $min^{-1}$ $g^{-1}$* | *Tumour flow ml $min^{-1}$ $g^{-1}$* | *Tumour: liver flow ratio* |
|---|---|---|---|---|---|---|
| 1 | 2.61 | 4.07 | 6.68 | 0.118 | 0.145 | 0.814 |
| | 2.64 | 5.24 | 7.88 | 0.107 | 0.149 | 0.72 |
| 2 | 9.61 | 9.15 | 18.76 | 0.447 | 0.767 | 0.58 |
| | 1.93 | 6.91 | 3.84 | 0.12 | 0.153 | 0.78 |
| 3 | 7.97 | 9.26 | 17.23 | 0.28 | 0.53 | 0.52 |
| | 1.56 | 5.08 | 6.62 | 0.048 | 0.105 | 0.45 |
| 4 | 10.47 | 7.48 | 17.55 | 0.272 | 0.939 | 0.29 |
| | 1.73 | 6.11 | 7.84 | 0.037 | 0.157 | 0.23 |
| 5 | 4.28 | 4.51 | 8.79 | 0.062 | 0.314 | 0.2 |
| | 4.27 | 5.1 | 9.37 | 0.147 | 0.3 | 0.49 |
| 6 | 4.81 | 5.45 | 10.26 | 0.125 | 0.36 | 0.34 |
| | 2.28 | 5.11 | 7.39 | 0.072 | 0.17 | 0.42 |
| 7 | 3.17 | 3.96 | 7.13 | 0.11 | 0.209 | 0.526 |
| | 1.67 | 5.75 | 7.42 | 0.085 | 0.102 | 0.83 |
| 8 | 6.39 | 7.67 | 14.06 | 0.3 | 0.41 | 0.73 |
| | 1.44 | 4.52 | 5.96 | 0.096 | 0.09 | 1.07 |
| 9 | 3.88 | 5.26 | 9.14 | 0.286 | 0.255 | 1.12 |
| | 4.08 | 6.65 | 10.73 | 0.38 | 0.266 | 1.43 |
| 10 | 3.73 | 4.73 | 8.46 | 0.312 | 0.386 | 0.808 |
| | 1.97 | 3.88 | 5.83 | 0.447 | 0.201 | 2.23 |

The first of each pair of values is the pre-infusion value and the second is the post-infusion value.

Hepatic arterial vasoconstriction takes place predominantly in the blood vessels of the normal hepatic parenchyma leading to an increase in the relative blood flow to hepatic tumour. This may be of clinical value as it may lead to preferential delivery of concomitantly administered cytotoxic agents to tumours with relative sparing of normal hepatic parenchyma.

In previous studies we have observed that SMS 201-995 inhibits the growth of hepatic tumour derived from the intraportal inoculation of Walker carcinosarcoma cells which was associated with a marked stimulation of the reticuloendothelial system (Nott *et al.*, 1989). Since the RES comprises the body's natural defence system against tumour cells, it was suggested that the stimulation of the Kuppfer cells may have been in part responsible for the inhibition of growth of the hepatic tumour. Although comparisons of liver blood flow were made between the two groups in that study, firm conclusions could not be made regarding the effect of SMS 201-995 on liver blood flow and its effect on tumour growth since there was very little tumour in the liver in the group treated with sandostatin at the end of the 3 week period. The results of this study however suggest that SMS 201-995, by reducing hepatic arterial flow may well influence the growth of hepatic tumour which derives its nutritive blood supply almost entirely from the hepatic artery.

This study was supported by the Cancer Research Campaign and the North West Cancer Research Fund. The HSN tumour was a gift of Dr S.A. Eccles, and SMS 201-995 a gift of Sandoz Pharmaceuticals.

### References

FINLAY, I.G., MEEK, D.R., GRAY, H.W., DUNCAN, J.G. & MCARDLE, C.S. (1982). Incidence and detection of occult hepatic metastases in colorectal carcinoma. *Br. Med. J.*, **284**, 893.

HEMINGWAY, D.M., COOKE, T.G., GRIME, S.J., NOTT, D.M. & JENKINS, S.A. (1991*a*). The effects of vasopressin infusion on hepatic haemodynamics in an experimental model of liver metastases. *Br. J. Cancer*, **64**, 212.

HEMINGWAY, D.M., COOKE, T.G., GRIME, S.J., NOTT, D.M. & JENKINS, S.A. (1991*b*). Changes in hepatic haemodynamics and hepatic perfusion index during the growth and development of hypovascular HSN sarcoma in rats. *Br. J. Surg.*, **78**, 326.

HEMINGWAY, D.M., COOKE, T.G., CHANG, D., GRIME, S.J. & JENKINS, S.A. (1991*c*). The effects of intraarterial vasoconstrictors on the distribution of a radiolabelled low molecular weight marker in an experimental model of liver tumour. *Br. J. Cancer*, **63**, 495.

JENKINS, S.A., BAXTER, J.N., CORBETT, W.A. & SHIELDS, R. (1985*a*). The effects of a somatostatin analogue SMS 201-995 on hepatic haemodynamics in the cirrhotic rat. *Br. J. Surg.*, **72**, 864.

JENKINS, S.A., BAXTER, J.N., CORBETT, W.A. & SHIELDS, R. (1985*b*). Effects of a somatostatin analogue SMS 201-995 on hepatic haemodynamics in the pig and on intravariceal pressure in man. *Br. J. Surg.*, **72**, 242.

KELLER, U., PERRUCHOUD, A., KAYASSEH, L. & GRY, N. (1978). Effect of therapeutic doses of somatostatin on splanchnic blood flow in man. *Eur. J. Clin. Invest.*, **8**, 335.

KEMENEY, N., DALY, J., REICHMAN, B., GELLER, N., BOTET, J. & ODERMAN, P. (1987). Intrahepatic or systemic infusion of fluorodeoxyuridine in patients with liver metastases from colorectal carcinoma. *Ann. Int. Med.*, **107**, 459.

KLEBER, G., SAUERBRUCH, T., FISCHER, G. & PAUMGARTNER, G. (1988). Somatostatin does not reduce oesophageal pressure in liver cirrhotics. *Gut*, **29**, 153.

KRAVITZ, D., BOSCH, J., ARDERIU, T., PIZCUETA, M.P., CASAMITJANA, R., RIVERA, F. & RODES, J. (1988). Effects of somatostatin on splanchnic haemodynamics and plasma glucagon in portal hypertensive rats. *Am. J. Physiol.*, **254**, G322.

MATTSON, J., APPLEGREN, L., KARLSSON, L. & PETERSON, M.I. (1977). Adrenergic innervation of tumour blood vessels. *Cancer Lett.*, **3**, 347.

MCDEVITT, D.G. & NIES, A.S. (1976). Simultaneous measurement of cardiac output and its distribution in the rat. *Cardiovasc. Res.*, **10**, 494.

NOTT, D.M., BAXTER, J.N., YATES, J., GRIME, S.J., DAY, D.W., COOKE, T.G. & JENKINS, S.A. (1989). Effects of somatostatin analogue (SMS 201-995) on the growth and development of hepatic tumour derived by intraportal injection of Walker cells in the rat. *Br. J. Surg.*, **76**, 1149.

PRICE, B.A., JAFFE, B.M. & ZINNER, M.J. (1985). Effect of exogenous somatostatin infusion on gastrointestinal blood flow and hormones in the conscious dog. *Gastroenterology*, **88**, 80.

SASAKI, Y., INAOKA, S. & MASEGAWA, Y. (1985). Changes in distribution of hepatic blood flow induced by intra-arterial infusion of angiotensin II in human hepatic cancer. *Cancer*, **55**, 311.

SIGURDSON, E.R., RIDGE, J.A. & DALY, J.M. (1986). Flurodeoxyuridine uptake by human colorectal hepatic metastases after hepatic artery infusion. *Surgery*, **100**, 285.

SONNENBERT, G.E., KELLER, U., PERRUCHOUD, A., BURCKHARDT, D. & GYR, N. (1981). Effect of somatostatin on splanchnic haemodynamics in patients with cirrhosis of the liver and in normal subjects. *Gastroenterology*, **80**, 526.


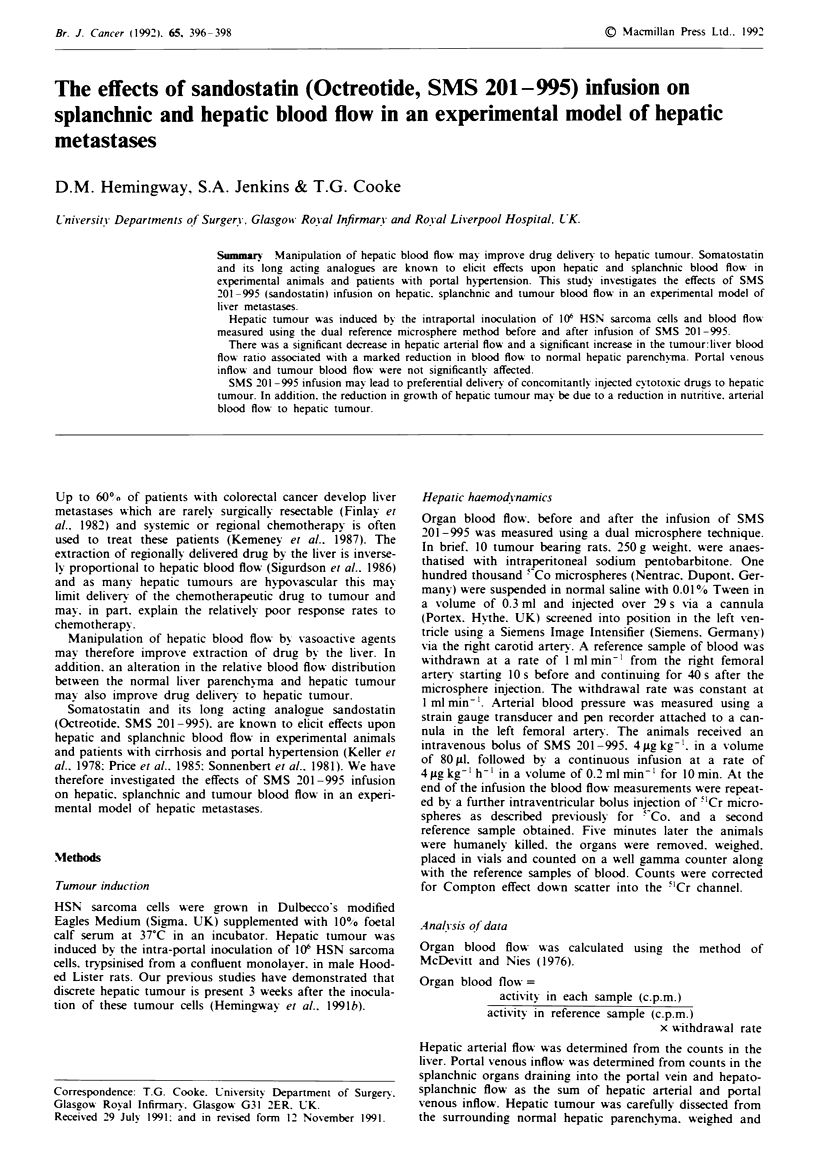

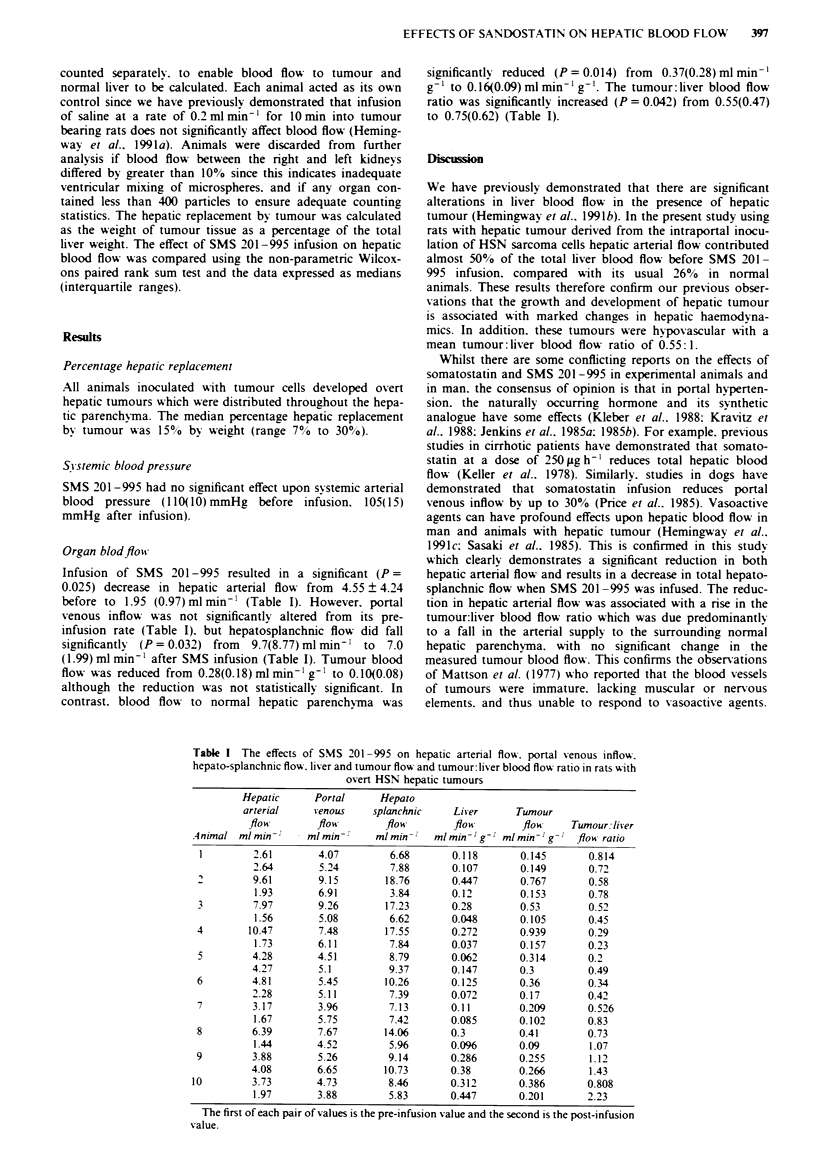

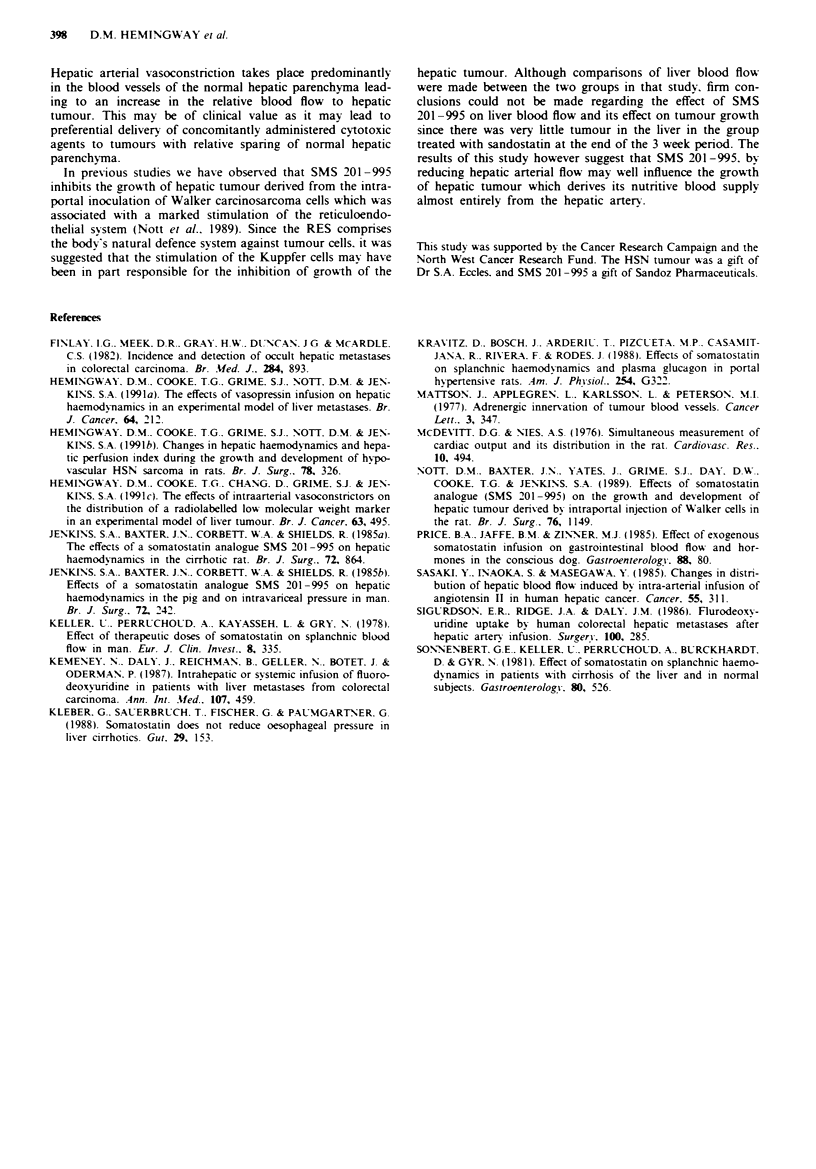


## References

[OCR_00308] Hemingway D. M., Chang D., Cooke T. G., Jenkins S. A. (1991). The effects of vasopressin infusion on hepatic haemodynamics in an experimental model of liver metastases.. Br J Cancer.

[OCR_00318] Hemingway D. M., Cooke T. G., Chang D., Grime S. J., Jenkins S. A. (1991). The effects of intra-arterial vasoconstrictors on the distribution of a radiolabelled low molecular weight marker in an experimental model of liver tumour.. Br J Cancer.

[OCR_00312] Hemingway D. M., Cooke T. G., Grime S. J., Nott D. M., Jenkins S. A. (1991). Changes in hepatic haemodynamics and hepatic perfusion index during the growth and development of hypovascular HSN sarcoma in rats.. Br J Surg.

[OCR_00323] Jenkins S. A., Baxter J. N., Corbett W. A., Shields R. (1985). The effects of a somatostatin analogue SMS 201-995 on hepatic haemodynamics in the cirrhotic rat.. Br J Surg.

[OCR_00339] Kemeny N., Daly J., Reichman B., Geller N., Botet J., Oderman P. (1987). Intrahepatic or systemic infusion of fluorodeoxyuridine in patients with liver metastases from colorectal carcinoma. A randomized trial.. Ann Intern Med.

[OCR_00345] Kleber G., Sauerbruch T., Fischer G., Paumgartner G. (1988). Somatostatin does not reduce oesophageal variceal pressure in liver cirrhotics.. Gut.

[OCR_00350] Kravetz D., Bosch J., Arderiu M. T., Pizcueta M. P., Casamitjana R., Rivera F., Rodés J. (1988). Effects of somatostatin on splanchnic hemodynamics and plasma glucagon in portal hypertensive rats.. Am J Physiol.

[OCR_00363] McDevitt D. G., Nies A. S. (1976). Simultaneous measurement of cardiac output and its distribution with microspheres in the rat.. Cardiovasc Res.

[OCR_00369] Nott D. M., Baxter J. Y., Grime J. S., Day D. W., Cooke T. G., Jenkins S. A. (1989). Effects of a somatostatin analogue (SMS 201-995) on the growth and development of hepatic tumour derived by intraportal injection of Walker cells in the rat.. Br J Surg.

[OCR_00378] Sasaki Y., Imaoka S., Hasegawa Y., Nakano S., Ishikawa O., Ohigashi H., Taniguchi K., Koyama H., Iwanaga T., Terasawa T. (1985). Changes in distribution of hepatic blood flow induced by intra-arterial infusion of angiotensin II in human hepatic cancer.. Cancer.

[OCR_00383] Sigurdson E. R., Ridge J. A., Daly J. M. (1986). Fluorodeoxyuridine uptake by human colorectal hepatic metastases after hepatic artery infusion.. Surgery.

[OCR_00388] Sonnenberg G. E., Keller U., Perruchoud A., Burckhardt D., Gyr K. (1981). Effect of somatostatin on splanchnic hemodynamics in patients with cirrhosis of the liver and in normal subjects.. Gastroenterology.

